# When Marriage Ends: Differences in Affluence and Poverty Among Older Adults in Israel

**DOI:** 10.1007/s10680-023-09676-1

**Published:** 2023-08-21

**Authors:** Alisa C. Lewin, Haya Stier

**Affiliations:** 1 Department of Sociology, University of Haifa, Haifa, Israel; 2 Department of Labor Studies, Department of Sociology and Anthropology, Tel Aviv University, Tel Aviv, Israel

**Keywords:** Marriage, Divorce, Affluence, Poverty, Gender

## Abstract

Studies show that the economic benefits of marriage carry over into old age and that widowhood and divorce have detrimental economic consequences, especially for women. This study asks how affluence and poverty are affected by the timing of widowhood and divorce and tests whether they operate in symmetry. The study draws on Israel’s annual Social Survey from multiple years (2013–2017), conducted by Israel’s Central Bureau of Statistics. The sample is limited to older individuals, aged 55+ (*n* = 4824 men, 5643 women). The findings show that married people are less likely to be poor than unmarried people, but they are not always more likely to be affluent. Widowed men and women, and divorced men are more likely to achieve affluence than continuously married couples. The explanation may be that, in the Israeli context, the widowed tend to inherit benefits accumulated by their late spouse, whereas the divorced tend to divide resources when the marriage dissolves. Women incur higher and longer-term penalties for their change in marital status than do men, so that previously married women tend to have higher rates of poverty and lower rates of affluence than previously married men. The findings show that affluence and poverty do not operate in symmetry and that affluence does not simply mirror poverty, especially among men. For example, early widowed and late divorced men have higher odds of both poverty and affluence than married men. These findings demonstrate that poverty and affluence operate differently and examining both leads to new insights.

## Introduction

The way a marriage ends has profound consequences on men and women’s economic well-being. Moreover, the *timing* of marital dissolution over the life course is likely to affect economic well-being later in life because the benefits of marriage carry over into old age, allowing older married couples to enjoy their joint accumulated assets (Brown & Wright, 2017; Waite & Gallagher, 2000; Zissimopolous et al., 2015). With the dramatic increases in longevity and divorce in the second half of the twentieth century, the ways marriages end have changed considerably. Widowhood still constitutes the majority of previously married older adults, but its share has declined and the share of divorcees among the older population has risen considerably (Brown & Lin, 2012; Hughes & O’Rand, 2004; Kreider & Ellis, 2011). Although most divorces occur at younger ages, scholars have observed an increase in late divorces (e.g., Brown & Wright, 2017), and the expression “gray divorce” has been coined to refer to divorces occurring after age 50. In general, widowhood takes place mostly later in life, and early widowhood is less common.

These demographic changes raise questions about the economic consequences of changes in marital status, because transitions out of marriage have been shown to affect economic well-being, especially among women (e.g., Andreß et al., 2006). In this study, we further argue that the economic consequences of marital dissolution depend not only on the transition out of marriage but also on how and when the marriage ends. Regarding how the marriage ends, we maintain that the consequences of divorce differ from those of widowhood, mainly because divorce entails more costs associated with the division of households. Widowhood is likely to be less economically detrimental than divorce because accumulated resources remain with the surviving spouse. As for timing, we argue that people who stayed married for a longer period are likely to have accumulated more resources, and hence, when marriage ends, they are likely to be better off financially than those who experience the dissolution at a younger age. The consequences of these life-course events are consequential to older adults' economic standing. The study also investigates gender differences, because the consequences of divorce and widowhood differ for men and women (Ahn, 2005; Andreß et al., 2006).

Many studies have looked at the detrimental effects of marital dissolution on economic well-being, focusing on declines in income and increases in poverty; but, to our knowledge, few have examined its effect on affluence (e.g., Hirschl et al., 2004, who compare affluence among married and non-married men and women). Indeed, scholars have noted that we know much more about poverty than about affluence (Iceland, 2019; Massey, 1996). Affluence is an important measure of inequality, with poverty and affluence being extremes of an income distribution that is becoming increasingly polarized. We maintain that focusing on both ends of the income distribution may contribute to our understanding of the mechanisms underlying the gendered economic effects of marital dissolution. For example, we know that divorced women are more likely to be poor than divorced men, but are they also less likely to be affluent? By focusing on older adults and on the long-term effects of transitions out of marriage, this study sheds light on processes generating economic inequality and on the economic welfare of vulnerable groups.

All in all, in this study we introduce five innovations. First, we distinguish divorce from widowhood. Second, we examine the effects of the timing of the transition into divorce and widowhood. Third, we look at the novel outcomes of affluence and poverty and fourth, we focus on older adults. Finally, we conduct the analyses separately for men and women so as to observe whether differences are gendered.

### The Israeli Context

The study was conducted in Israel, a country with a pro-family orientation reflected in near-universal marriage and high fertility, primarily within marriage (Okun, 2013). Although they have risen in recent decades, divorce rates are low when compared to most industrialized countries (Total Divorce Rate 26–27% in Israel vs. 35% in EU countries) (Nahir, 2016). Due to its high fertility rate, Israel’s population is relatively young even though the share of the adult population age 55 and older has increased from 15.8% in 1990 to 19.9% in 2015. Similar to many industrialized countries, life expectancy has increased for both men and women (from 74.9 to 80.7 for men and 78.4 to 84.1 for women, between 1990 and 2015, respectively) (CBS Israel, 2016). Among the population 65 and older, 10% of men were divorced, about 10% were widowed, and about 3% were never married. Among women 65 and older, 13.6% were divorced, 15% were widowed, and 4.5% were never married (CBS, 2020). Rates of non-marital cohabitation are increasing, but mainly among young never-married individuals, and cohabitation is less common among older adults (Bystrov, 2012).

Earnings constitute the major source of income for the working age population and, to some extent, for those reaching retirement age, although most of the elderly in Israel are supported by social security and pension systems. The social security program consists of old-age allowances and survivor benefits, which are universal after retirement age (with some exceptions); the pension system is comprised of occupational pensions that are income-dependent (Lurie, 2018a, 2018b). The sum of social security benefits is low when compared to OECD countries and does not provide adequate income after retirement (NII Website, 2020; OECD, 2010, p. 188). Residents without additional income are entitled to a supplemental income allowance in addition to the old-age allowance. The sum of the two equals about $900 monthly (NII website, 2020), which just about reaches the poverty line. In the past, widows in Israel (as in several other countries) were entitled to different rights than widowers in the state pension system as well as in occupational pension arrangements. With time, several reforms have been implemented to equalize the rights of widows and widowers (Estelle, 2009).

Occupational pensions play an important role in providing income security for Israeli retirees, due to the relatively low level of old-age allowances. Occupational pensions are linked to the salaries earned near retirement and to savings plans (involving contributions from both workers and employers). Occupational pensions were made mandatory in Israel as recently as 2008 (Gavious et al., 2009), so that currently, about two-thirds of retirees in Israel have access to such plans (Toledano & Gottlieb, 2014).

In Israel, marriage and divorce are controlled by religious law. Divorce (for Jews) is granted solely by rabbinical courts, and post-separation arrangements (including child custody, alimony and the division of assets) may also be determined by civil courts (Hacker, 2008). Divorce legislation is based on the assumption that women are responsible for reproduction and raising children (Halperin-Kaddari, 2007; Triger, 2005); hence, children typically remain under the custody of mothers. This affects the economic status of single-mothers, who depend primarily on income from work because they receive relatively low levels of State support (Stier, 2011). We argue that the economic difficulties divorced Israeli women experience (e.g., Kaplan & Herbst, 2015; Lewin & Stier, 2018; Raz-Yurevich, 2013) have long-term effects that carry over into old age, also in the form of low pensions. The law was amended in the 1990s, so that pensions accumulated during marriage are divided between parties upon divorce (Gaifman, 1992; Lifshitz, 2016), although studies show that women still encounter difficulties enforcing the rulings (Lurie & Stier, 2022).^1^

### The Importance of Distinguishing Divorce and Widowhood Later in Life

The ending of marriage is associated with economic vulnerability, as married individuals enjoy more financial security than do previously married individuals, both divorced and widowed (Ahn, 2005; Bíró, 2013; Lin et al., 2017; Waite & Gallagher, 2000). Furthermore, the long-term economic consequences of divorce, especially for women, are also well-documented (Andreß et al., 2006; Dewilde & Stier, 2014; Jansen et al., 2009; Lewin & Stier, 2018). In this study, we adopt a life-course approach and we focus on the long-term economic consequences of marital dissolution, taking into account both *how* and *when* the marriage ends.

Indeed, most prior research on the economic consequences of marital dissolution has focused on changes in economic position following divorce and widowhood, but relatively few studies have taken into account the effect of the timing of these events and none have focused on affluence and poverty. We argue that the economic consequences of divorce at older ages may differ from divorce at younger ages because it is not likely to involve young children who require custody arrangements or intense caregiving. In parallel, although widowhood usually occurs at older ages, there is a small portion of the population that becomes widowed at younger ages, before assets can be accumulated and pensions accrued. The economic consequences of younger widowhood are therefore likely to differ from those of widowhood later in life. Most studies that have examined the economic consequences of divorce and/or widowhood have concluded that the two events detrimentally affect partners’ economic well-being and that these effects are gendered (Ahn, 2005; Andreß et al., 2006; Manting & Bouman, 2006; Tach & Eads, 2015). In brief, the financial well-being of widowed people depends to a great extent on the pensions they and their partners accrued, the insurance policies they purchased, and the resources they accumulated, all of which are likely to differ substantially by income, age, and gender. Welfare policy that allocates allowances and survivor benefits also affects widows’ and widowers’ financial situation.

It should thus be clear that divorce and widowhood do differ in their consequences—even though both entail a transition from a two-headed household with two potential earners to a single-headed household with perhaps only one earner. Divorce may be more harmful economically because family assets must be divided between two newly formed households**.** In contrast, in the case of widowhood, the surviving spouse typically inherits control over assets accumulated during the marriage. These differences may vary, depending on the timing of the event. For instance, when dissolution occurs early in life, widowhood may be even more harmful than divorce because it is likely to be unexpected and therefore devoid of any arrangements regarding the pair’s economic assets. In contrast, people anticipating divorce may find ways to reduce its damages in advance and may even time the divorce to minimize its harmful economic implications. Widowhood at young ages is also likely to affect young dependent children who, unlike the case of divorce, cannot benefit from the financial and emotional support of the non-resident parent (Amato & Anthony, 2014; Özcan & Breen, 2012).

Studies show that economic differences between married and previously married individuals are greater at older than younger ages because the married (and the remarried) managed to accumulate more assets and achieve higher earnings during their years of marriage than the divorced and widowed (Zissimopolous et al., 2015).

Re-partnering may offset economic declines following divorce and widowhood because spouses tend to pool resources in marriage (Dewilde & Uunk, 2008); however, the likelihood of re-partnering differs by gender and age. Studies show that men re-partner more often and faster than do women after divorce and widowhood (De Graaf & Kalmijn, 2003; Ivanova et al., 2013; Wu & Schimmele, 2005), and that the gender gap in remarriage appears at all ages (Wu et al., 2014).

### Economic Consequences, by Gender

The economic consequences of how and when a marriage ends are likely to differ by gender. Numerous studies have shown that divorce entails a more severe decline in economic resources for women than for men (Andreß et al., 2006; Manting & Bouman, 2006; Tach & Eads, 2015). Widowhood is also associated with declines in economic well-being that are, again, more detrimental for women than for men (Ahn, 2005; Angel et al., 2007). Older women are also more likely to be widowed than are men because they tend to outlive their spouses.

Gender differences in the detrimental effects of marital dissolution result, to a great extent, from the inter-dependence of spouses in marriage and from the unequal division of labor within the family (Hobson, 1990; Korpi, 2000; Millar & Ridge, 2009). Wives are often economically dependent on their partners because women typically earn less than men and they tend to have more disrupted work patterns because of parenting responsibilities (Sorensen & McLanahan, 1987; Stier & Mandel, 2009; Stier et al., 2018). Women also tend to gain custody over young children following divorce, thus they tend to have larger households than divorced men. Hence, women usually have more difficulties than do men in overcoming the loss of their partners’ income following marital dissolution (McManus & DiPrete, 2001; Raz-Yurevich, 2013).

A vast literature documents gender differences in the economic consequences of marital breakdown and demonstrates the ways in which women become economically vulnerable following marital disruption. For example, previously married women have higher odds of living in poverty and experiencing economic hardship than do married women (Lewin & Stier, 2018) and lower odds of achieving affluent incomes than do married women (Hirschl et al., 2004).

Although men may also suffer economic losses following divorce (McManus & DiPrete, 2001), most studies show that women experience sharper declines in income than do men and that women suffer from higher rates of poverty and more severe deterioration in living conditions (Andreaß et al., 2006; Dewilde, 2009; Lewin & Stier, 2018). Moreover, while men recover relatively quickly from the hardships following marital breakup, these detrimental effects tend to be long term among women (Dewilde & Stier, 2014; Lewin & Stier, 2018).

Gender differences in the economic outcomes of marital dissolution may be affected by the timing of the divorce and widowhood over the life course. First, divorce and widowhood later in life are not likely to involve young children. This absence of young children implies fewer gender differences in custody arrangements, which may affect economic consequences and re-partnering patterns and thereby reduce the costs of divorce for these women. However, because of their previous interrupted employment while caring for children, older women generally accumulate lower pensions and less job experience; they subsequently have fewer opportunities to find employment later in life (Stier & Endeweld, 2015). The relatively few studies on gray divorces indicate greater penalties for women than for men (Crowly, 2018) because disadvantages accumulated over the life course carry over into later adulthood. Gender differences in the consequences of later divorce and widowhood reflect cumulative disadvantages whereas at younger ages, disadvantage may reflect the unequal burdens of caregiving to children.

These gender differences are compounded by the differential likelihood of remarriage. Fathers are more likely to re-partner than are mothers (Ivanova et al., 2013); hence, fathers tend to remain in the “divorced” or “widower” state for shorter periods than do mothers. Divorced and widowed mothers may delay transitioning into a new marriage either because they are not perceived to be attractive mates or because they do not desire entry into a new marriage while caring for young children (Mahay & Lewin, 2007; Wu et al., 2014). In addition, a simple duration effect comes into play: The younger the age at divorce or widowhood, the more time a person has to re-partner.

The probability of remarriage also declines with age as the result of the smaller supply of unmarried potential partners at older ages. This constraint in the marriage market favors men because there are more widows than widowers available due to gender differences in longevity. There is then, a selection into remarriage, and this selection is related to gender and age in addition to economic resources. In this study, we compare all marital status groups, including the small “never-married” group. People who never married never benefitted from the pooling of resources and the utilities of division of labor, but they also never had to suffer the cost of a breakup. This makes it interesting to examine this group’s economic standing later in life.

### Poverty and Affluence in Older Age

Many studies have examined the economic consequences of marital dissolution, focusing on household income and on poverty, but few have looked at affluence. Indeed, much more is known about poverty than affluence (Iceland, 2019; Massey, 1996). One reason for this imbalance is that poverty is defined as a social problem associated with a host of negative outcomes that require policy attention, whereas affluence is a desired status, with positive effects on education, opportunity, and accumulation of assets (Zissimopolous et al., 2015). Consequently, countries have adopted an official “poverty line” to register poverty, account for fluctuations, and to draw policymakers’ attention to where change is needed. A parallel “affluence line” has not been developed, perhaps because it does not reflect hardship and therefore does not invite any government intervention requiring the mobilization of public resources.

Older adults in poverty are at risk of experiencing hardships due to increased expenses in health management and care, whereas affluent older adults are better prepared for their changing health needs and expenses. Affluence at older age may translate to having access to costly health services that are not covered by insurances, as well as to being able to relocate to high quality assisted living housing arrangements, when needed. Moreover, affluence among older adults is related to better quality of life, more comfortable living arrangements, and better time use during retirement. Affluent older adults also have resources to transmit to their children and grandchildren. In sum, affluence and poverty may have far-reaching implications on well-being later in life, and this study’s results may inform policies aimed at reducing hardship among older adults.

Studies show that poverty and affluence usually operate in symmetry, so that factors associated with increased affluence tend to reduce poverty, and factors that increase poverty tend to reduce affluence (Iceland, 2019; Lewin, 2022). Violations of this symmetry may reveal policy intervention as well as differences in group characteristics (Iceland, 2019). For example, affluence might be affected by pension regulations and social benefits that are not uniformly distributed across social groups, whereas poverty is affected by welfare policy and eligibility criteria for income supplements and grants.

### Research Hypotheses and Analytical Framework

In light of previous research on consequences of marital dissolution, we expect that the continuously married will experience more affluence and less poverty than those who experienced a marriage breakup or those who never married. We also expect the economic outcomes of transitions out of marriage to be more detrimental for women than for men because women earn less than men, on average, they are less likely to be involved in paid employment at older ages and their own pension endowments are usually smaller than that of men.

Our study focusses on the effects of how and when marital dissolution happened. Grounded in the literature on marriage transitions among the older population, the specific arrangements in Israel regarding the rights of those divorced and widowed, and our interest in their effect on affluence and poverty we constructed four hypotheses:

#### H1

We expect early divorce and widowhood to be associated with more poverty and less affluence than late divorce and widowhood because they are likely to involve young children and more expenses. Moreover, older couples have had time to accumulate more resources, pension rights and benefits than young couples and they are likely to be in a better economic position when the dissolution occurs.

#### H2

We expect the economic outcomes of divorce to be more detrimental than the economic outcomes of widowhood because the widowed benefit from resources accumulated during marriage and the late spouses’ pension funds, whereas the divorced do not have full access to spouses’ funds and pensions and accumulated assets are divided.

#### H2(a)

We expect gender differences to be smaller among the widowed than the divorced because widowed women have better access to accumulated resources and husbands’ pensions than divorced women. Furthermore, gender differences are expected to be greater for early transitions out of marriage than later transitions because fewer resources had been accumulated before dissolution.

#### H3

We expect the never married to have less affluence and more poverty than the married and the widowed. However, we expect the never married to have more affluence and less poverty than the divorced because they are mostly without family responsibilities and their accumulated resources never had to be divided.

#### H4

We expect to find symmetry in affluence and poverty, so that groups that are over-represented among the affluent will be under represented among the poor.

### Data and Measurement

The study draws on Israel’s Social Survey from multiple years (2013–2017), conducted by its Central Bureau of Statistics. The Social Survey is a representative sample of Israeli households whereby one individual is selected as a respondent for the survey. Our sample includes respondents aged 55 and above, and we compare men and women from different households, not husbands and wives from the same household. The data were collected in face-to-face interviews conducted in Hebrew, Russian, and Arabic, using CAPI. The sample includes respondents in assisted living housing, but does not include people in nursing homes or in hospitals. The total sample included 4,067 men and 4430 women. Despite the fact that the Social Survey is a cross-sectional survey, it is suitable for our investigation thanks to its retrospective questions on the timing of marital transitions and its large sample size that allowed us to compare seven groups, by gender. Arab citizens of Israel were excluded from the current study due to the very low divorce rate in these communities (Lewin, 2006).

#### Dependent Variables

Our dependent variables capture the two extremes of the income distribution: poverty and affluence. Both measures are equivalized, which means they take account of the number of people in the household.^2^ Our measure of poverty emulates Israel’s official poverty line, which is based on the net median household income per capita. As there is no official “affluence line,” we chose to measure affluence in reference to the same net median household income per capita we use for measuring poverty. Poverty and affluence thresholds were computed for each year for the entire sample included in the social survey data (aged 20+) and then applied to our sample of Jewish older adults.

*Poverty* is a dichotomous variable indicating whether net household income per capita is equal to or below half of the net median household income per capita. The percentage of the population living in poverty according to this measure is similar to the percent living in poverty according to Israel’s official measure of poverty (NII, 2016), indicating that this is a reliable measure.

*Affluence* is a dichotomous variable indicating whether net household income per capita is equal to or higher than twice the net median household income per capita. We acknowledge that other measures of affluence exist (see Iceland, 2019, for a recent review), but we chose the measure based on the median to parallel our measure of poverty.

#### Independent Variables

The main independent variable is a composite measure of marital status, by timing. We especially want to distinguish late (“gray”) divorce from early divorce and early widowhood from later widowhood. We compare seven groups: (1) early marriage (married before age 50^3^); (2) late marriage (married at age 50 and after); (3) early divorce (divorced before age 50); (4) late divorce (divorced at age 50 and after); (5) early widowhood (widowed before age 50); (6) late widowhood (widowed at age 50 and after); and (7) never married. For consistency with divorce and widowhood, we define marriages by age rather than by parity and we do not distinguish between first and higher-order marriages. We acknowledge that there are a small number of early remarriages and of late first marriages, and we know that most late marriages are, in fact, higher-order marriages. Rates of cohabitation among older adults in Israel are low (Bystrov, 2012), which may explain why information on non-marital cohabitation was not collected in the social survey. Nonetheless, we acknowledge that unmarried individuals may be living with a partner. As explained earlier, our dependent variables are adjusted by household size, which addresses part of this issue.

Our statistical models control for characteristics related to economic outcomes: age (in years), education (four categories of highest educational level achieved: less than high school; high school; post-secondary; academic—reference category in regressions), participation in paid employment in the survey year, number of children ever born (ranging from 0 to 7), and self-reported health status (ranging from 1 = very good to 4 = not good at all). We include indicators for immigration status (whether respondent immigrated to Israel from the former USSR = 1; otherwise = 0), and whether the respondent is religiously observant (ultra-Orthodox = 1: otherwise = 0). Because we pooled data for five years (2013–2017), we also control for survey year. Logistic regressions were computed to predict poverty and affluence. All analyses were conducted separately, by gender, using Stata 15. Missing cases were deleted listwise.

### Findings

Table 1 presents descriptive statistics, by gender. Starting with the dependent variables, Table 1 shows that on average, a slightly higher percentage of women are poor when compared to men (8.7% and 7.0%, respectively). In parallel, a higher percentage of men than women report affluent incomes (30.1% and 25.2%, respectively). In sum, this sample of older Jewish adults in Israel is better off financially than the general population, where about 20% are to be found below the poverty line, and about 20% above the “affluence line” (Lewin, 2022).

**Table 1:** Means (standard deviations) and percentage distribution, by gender *Source*: Israel Social Survey, 2013–2017

	Men	Women
*Dependent variables*		
% Poor	7.0	8.7
% Affluent	30.1	25.2
*Independent variables*		
% Married young	77.5	56.6
% Married late	4.5	2.1
% Divorced young	4.6	9.2
% Divorced late	4.8	4.7
% Widowed young	0.5	4.4
% Widowed late	5.8	19.0
% Never married	2.4	4.0
Age (mean + SD)	67.47 (8.80)	68.27 (9.08)
*Education*		
Less than high school	17.3	18.9
High school	25.6	26.8
Post-secondary (non-academic)	23.5	22.7
Academic	33.6	31.7
% Immigrated from the USSR after 1990	23.2	28.6
% Ultra-orthodox	4.6	3.3
% Work	53.5	36.1
Number of children ever born (mean + SD)	2.97 (1.50)	2.72 (1.56)
Health status (mean + SD)	2.16 (0.86)	2.32 (0.89)
N	4067	4430

Poverty was measured as below half the median per capita net household income; affluence was measured as twice the median per capita net household income

The second panel of Table 1 shows that most men and women aged 55 and above were in long-term marriages that began at a young age, but the percentages are significantly higher for men (77.5%), than for women (56.6%). This may be explained by the fact that women are more likely than men to transition into late widowhood because they live longer than do men, on average. A small minority of respondents had married after age 49, but these are most likely higher-order marriages. The figures show that late marriage is more prevalent among men than women, which corresponds with findings on gender differences in rates of remarriage. Overall, at the time of the survey, over 80% of men, but fewer than 60% of women, were married. Fewer than 10% of men were divorced at the time of the survey, of whom about half divorced at a young age (and did not remarry), and half divorced after age 50. The numbers are somewhat different for women, of whom 9.2 percent divorced young and stayed divorced and 4.7% divorced late. A small minority of men and women lost their spouses prior to age 50, while almost fifth of all women (19%) and 5.8% of all men were widowed at an older age. Lastly, 2.4% of men and 4% of women in our sample never married.

Men and women were found to be similar in their average age, level of educational attainment, and immigration status. A higher percentage of men than women participate in paid employment (53.5% among men compared to 36.1% among women).

We now turn to our multivariate analyses. We set out to ask how the timing of the transition out of marriage affects poverty and affluence, by gender. Table 2 presents logistic regression coefficients predicting the odds of poverty and affluence, by gender. Beginning with poverty, Table 2 shows that the odds of being poor are affected by transitions out of marriage and in some cases, also by the timing of the transition.^4^ First, as expected, marriage is associated with lower odds of being poor, regardless of timing, for both men and women. As expected, divorce (independent of timing) increases the odds of being poor. However, tests of differences between coefficients show that the differences between young and late divorce are statistically insignificant for men and borderline significant (*t* = 1.83) for women.

**Table 2:** Regression coefficients (standard errors) predicting poverty and affluence, by gender *Source*: Israel Social Survey, 2013–2017

	Poverty		Affluence	
	Men	Women	Men	Women
*Marital status*				
Married late	0.370 (0.325)	0.547 (0.426)	− 0.068 (0.188)	0.038 (0.268)
Divorced young	0.897* (0.259)	1.463* (0.172)	− 0.369* (0.181)	− 0.197 (0.149)
Divorced late	0.555* (0.282)	0.918* (0.242)	1.119* (0.165)	0.069 (0.191)
Widowed young	1.433* (0.632)	0.453 (0.275)	1.107* (0.521)	0.482* (0.194)
Widowed late	0.264 (0.276)	0.582* (0.168)	0.421* (0.173)	0.356* (0.121)
Never married	1.683* (0.350)	1.256* (0.297)	− 0.137* (0.268)	− 0.217 (0.209)
Age	− 0.063* (0.009)	− 0.032* (0.008)	0.042* (0.006)	0.047* (0.006)
*Education*				
Less than high school	1.269* (0.215)	1.263* (0.196)	− 1.975* (0.140)	− 2.030* (0.148)
High school	0.759* (0.209)	0.671* (0.185)	− 1.275* (0.102)	− 1.167* (0.102)
Post-secondary (non-academic)	0.135 (0.226)	0.459* (0.186)	− 0.724* (0.099)	− 0.620* (0.105)
Immigrated from the USSR after 1990	0.537* (0.187)	0.409* (0.148)	− 1.680* (0.115)	− 2.035* (0.124)
Ultra-orthodox	1.198* (0.235)	1.029* (0.247)	− 0.853* (0.238)	− 0.604* (0.255)
Work	− 1.325* (0.168)	− 1.024* (0.163)	0.659* (0.098)	0.564* (0.099)
Number of children ever born	0.309* (0.045)	0.187* (0.039)	− 0.203* (0.033)	− 0.196* (0.035)
Health status	0.299* (0.080)	0.601* (0.073)	− 0.369* (0.053)	− 0.640* (0.056)
Year of survey	0.006 (0.047)	0.035 (0.040)	0.026 (0.027)	0.015 (0.028)
Constant	− 0.619 (0.972)	− 1.723 (0.846)	− 2.079 (0.586)	− 1.701 (0.591)
Pseudo *R*^2^	0.176	0.154	0.163	0.197

Poverty was measured as below half the median net per capita household income; affluence was measured as twice the median per capita net household income

*p < 0.05

Widowhood also increases the odds of being poor, and these effects differ by timing. Young widowhood increases the odds of poverty for men, whereas late widowhood does not. Among women, however, late widowhood increases the odds of being poor, whereas young widowhood does not. These findings provide partial support for H1 and show that the effects of the timing of widowhood on poverty differ by gender. Although divorce (early and late) and late widowhood are generally more detrimental for women than for men, gender differences in odds of being poor are not statistically significant. The gender difference in young divorce is borderline significant (*t* = 1.82).

The study’s findings indicate that in most cases, widowhood is less detrimental than divorce, with the exception of young widowhood among men, providing partial support for H2. We find partial support for H2a in regard to poverty because we find some gender differences are only borderline statistically significant and others are non-significant. Our findings do support H3, that never married men and women are at higher risk of being poor when compared to those who married early (*b* = 1.68 for men and *b* = 1.26 for women). These highlight the long-term effects of pooling resources in building economic resilience and avoiding poverty among older married adults.

The second panel of Table 2 (columns 3 and 4) shows logistic regression coefficients predicting the odds of being relatively affluent (that is, twice the net median income per capita). Contrary to our expectations (H4), these findings are complex, showing that affluence is not always a mirror image of poverty. For example, the continuously married have lower odds of poverty than most other groups, but they do not have higher odds of affluence than most other groups. Some groups are very diverse and are over-represented at both ends of the income distribution. For example, early widowed men and late widowed women have higher odds of both poverty and affluence than the continuously married.

These findings suggest that, among men, widowhood (both young and late) and late divorce do not significantly interfere with the ability to achieve affluence despite these transitions out of marriage. Among men, the difference between young and late divorce is statistically significant, with late divorce being associated with higher odds of affluence than early divorce, as hypothesized (H1). However, the findings do not support H2, and late divorce is more beneficial for men than late widowhood (H2).

The results show that, contrary to our expectations, transition out of marriage does not seem to affect women’s odds of affluence. Widowed women have higher odds of affluence than continuously married women, and the differences between divorced and continuously married women are not statistically significant. Women who widowed early in life have higher odds of being affluent than those divorcing at a younger age, as we hypothesized.

The gender differences in the odds of poverty are not statistically significant, maybe because these are relatively small groups and poverty rates are low in this population. But, gender differences in the odds of affluence among the divorced are statistically significant, suggesting that divorce is more detrimental to women than men. Gender differences among the widowed are not statistically significant, suggesting that survivor benefits and pension policies are successful in protecting women.

As for the control variables, men and women who work for pay have lower odds of being poor and higher odds of being relatively affluent. In addition, those with health issues, ultra-Orthodox Jews, and immigrants from the former USSR have higher odds of being poor and lower odds of being affluent. The effect of education on the odds of being poor and being affluent is monotonic—people with an academic degree (reference category) have the lowest odds of being poor and highest odds of being affluent while those who did not complete high school have the highest odds of poverty and lowest odds of affluence. The differences between all educational groups are statistically significant.

## Predicted Marginal Probabilities

**Fig. 1:**
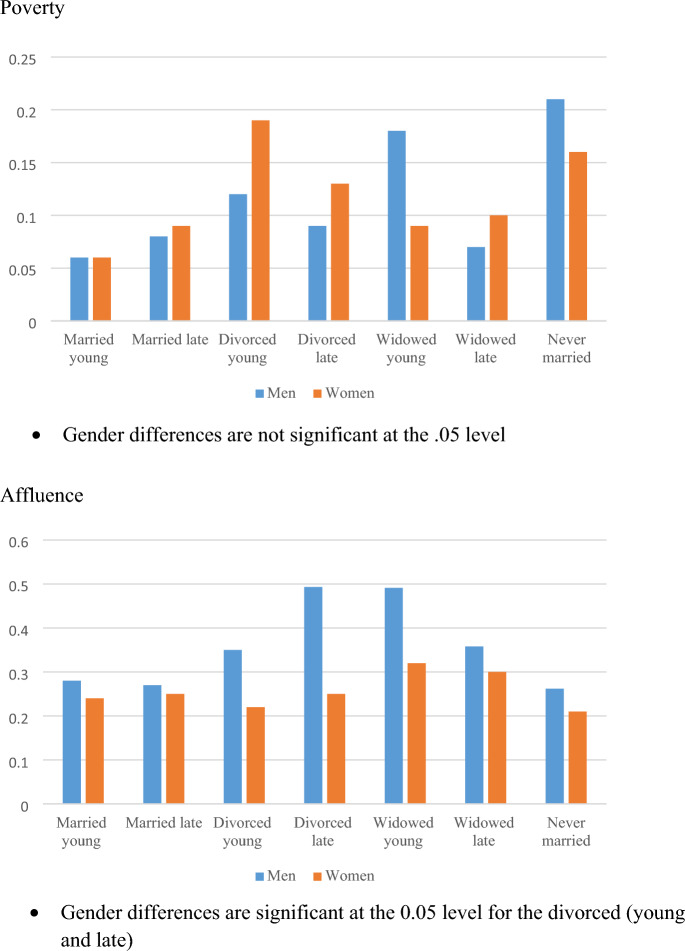
Predicted marginal probabilities of poverty and affluence, by timing of transition out of marriage and gender (based on Table 2)

To summarize the effect of marital status and the timing of transition out of marriage, we turn to predicted marginal probabilities. Figure 1 presents the marginal probabilities of poverty and affluence, by marital status and timing of the transition, for men and women. The figure depicts gender differences. With two exceptions (divorced young and never married), women have higher predicted probabilities of being in poverty than men, and in no case do women have higher predicted probabilities of affluence than men. In fact, divorced men have higher probabilities of affluence than divorced women, and the differences are statistically significant. Although late married women appear to have higher predicted marginal probabilities of poverty than late married men, the difference is not statistically significant.

## Discussion

Our study examined poverty and affluence among older adults in Israel and asked how they are associated with transitioning out of marriage. Grounded in a life-course approach, the study focused not only on the different types of transition out of marriage but also on the timing of those marital events. As expected, we found that married people are less likely to be poor than are most unmarried people, and we found no differences between early and late marriages in poverty or in affluence.

We found that timing matters and that people who transition out of marriage early in life are at higher risk of poverty and do not seem to close the gap with those who remain married.

How a marriage ends seems to matter even more than timing, and (with the exception of early widowed men), divorce tends to be more economically detrimental than widowhood, for men and women, as expected. Widows benefit from their spouse’s pensions and joint savings, so they have higher odds of affluence than the married. However, widowed late women also have higher odds of poverty, because not all spouses had occupational pensions.

We also found gender differences in the likelihood of being affluent, whereby divorced women are less likely to be affluent than previously married men. This aspect of gender inequality would not have been exposed had we not focused on extreme measures of poverty and affluence. The under-representation of unmarried women in affluence may not appear to be an acute social problem as is the over representation of unmarried women in poverty, found in other studies, but we argue that it is an important indicator of inequality. It also affects the conditions in which children in female-headed homes are raised and the opportunities open to them. Studies also show that mothers are more involved in intergenerational transfers to adult children than fathers, so that the funds available to mothers have lasting effects that may span generations (Clark & Kenney, 2010; Ophir, 2012).

The gendered mechanisms related to remarriage underlie some of the differences we observe. Divorced and widowed men with few economic resources may have lower odds of remarrying or may take longer to remarry than men with more resources. Women with more resources may be attractive candidates for remarriage, but they may have less of a desire to remarry than men may have, especially if they have children (Lewin, 2018; Mahay & Lewin, 2007), which may lead to lower rates of remarriage. Some pension policies may impose a disincentive to remarry because widows may lose their pension benefits upon remarriage. It is therefore important to trace not only the timing of the event but also the entire marital history in order to understand who remarries and how quickly they transition into (re)marriage after marital breakup. We acknowledge that many of those who married at an older age (after 49) are probably in higher-order marriages but, unfortunately, with our cross-sectional data, we could not account for selection into (re-) marriage. We did control for variables that may affect such selection, which may reduce the bias, but we acknowledge we cannot account for selection.

There is also a selection into first marriage, which leaves the “never married” as a unique group in a potentially vulnerable economic position. The never married could never benefit from the pooling of resources but, at the same time, most had no responsibilities associated with raising children. Although the never married may be cohabiting, prevalence of long-term non-marital cohabitation in Israel is a relatively new phenomenon, and rates among the older population are low (Bystrov, 2012). We would expect the never married in the selected age group to be better off than the divorced because in most cases they never paid the penalty of splitting resources, and experienced no work interruptions due to childbearing, especially in the case of women. However, we do find that the never married differ from the divorced, and in most cases their economic position is even worse. This might indicate, again, the selectivity of this group in a society characterized by near-universal marriage.

This study has some limitations. First, we use cross-sectional data to investigate a life-course question, which by nature is longitudinal. Our dependent variables are based on income, which may fluctuate from year to year. Nonetheless, in the case of older adults, who rely on pensions and allowances, (which tend to be fixed or linked to the cost of living index) this may be less of an issue than for the working aged population. Another limitation is that our sample does not include people in nursing homes and hospitals, thereby excluding people in the worst health status.

This study has important implications for theory and for policy. First, how a marriage ends matters (divorce being more detrimental than widowhood), partly because divorce is more costly as assets are divided between spouses, but also because of how pensions and allowances are allocated. In the Israeli case, while the widowed enjoy the benefits (or parts of the benefits) accumulated by their late spouse, this is not the case for the divorced. Policy arrangements should clearly define access to ex-partner’s pensions accumulated during marriage. Moreover, they should take into account women’s duties as (unpaid) caretakers in calculating their pension savings and allowances, in order to improve their economic standing when marriage dissolves.

Second, timing matters as well, and people whose marriage dissolves at a young age experience more poverty than people who remained married continuously. This has important implications on health, well-being, and living arrangements in old age. Finally, this study highlights the importance of examining affluence and poverty. More specifically, the findings contribute to a better understanding of gender inequality along the life course as we found profound gender differences in affluence among the divorced. Although women’s under-representation in affluence may not be considered a central social problem as is poverty because it is not associated with severe material hardship, it is an important indicator of gender inequality. Moreover, we maintain that focusing on affluence may reveal ways in which gender inequality is generated and reproduced. We conclude that our study opens a new research agenda for investigating dynamics of gender inequality by focusing on the extremes of the income distribution and examining poverty and affluence and how they change over the life course.

## Footnotes

1 In 2014, Israel's Parliament legislated a law that specifies the ways court rulings in cases regarding division of pension assets should be applied.

2 We use the NII’s equivalency table for weighting household size when calculating the official poverty line (NII, 2016).

3 We use age 50 as an age threshold distinguishing early and late transitions into divorce and widowhood. We acknowledge that this threshold is not early for transition into marriage, but we apply it nonetheless to maintain comparability with the other transitions, which are the focus of the analysis.

4 We note that despite the inclusion of many variables, the majority of the variation remains unexplained.
